# (*E*)-2-{[1-(3,11-Dimethyl-4-methyl­ene-10-oxo-1-phenyl-4,5,10,11-tetra­hydro-1*H*-benzo[*b*]pyrazolo­[3,4-*f*][1,5]diazo­cin-5-yl)ethyl­idene]amino}-*N*-methyl-*N*-(3-methyl-1-phenyl-1*H*-pyrazol-5-yl)benzamide

**DOI:** 10.1107/S1600536813025671

**Published:** 2013-09-25

**Authors:** Fiorella Meneghetti, Benedetta Maggio

**Affiliations:** a Department of Pharmaceutical Sciences, University of Milano, via L. Mangiagalli, 25, 20133-Milano, Italy; b Dipartimento di Scienze e Tecnologie Biologiche, Chimiche e Farmaceutiche, University of Palermo, via Archirafi, 32, 90123-Palermo, Italy

## Abstract

The central eight-membered ring of the title compound, C_40_H_36_N_8_O_2_, deviates from the ideal boat conformation because the bond between the *exo*-ethyl­ene group and the adjacent N atom is twisted by 60.0 (4)° due to steric hindrance. Its adjacent benzene and pyrazole rings are oriented almost perpendicular to each other, making a dihedral angle of 85.8 (3)°. In the crystal, the mol­ecules are linked by C(ar)—H⋯O hydrogen bonds, generating a three-dimensional network.

## Related literature

For the synthetic method, see: Plescia *et al.* (1979 ▶, 1983 ▶). For background to the bioactivity of benzo­diazo­cine derivatives, see: Milkowski *et al.* (1985 ▶); Heitmann *et al.* (1988 ▶).
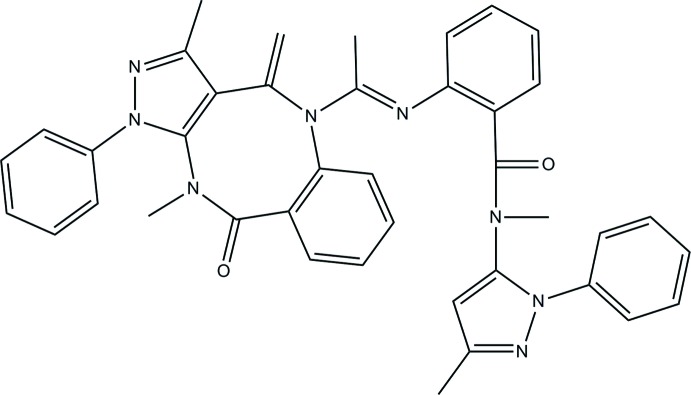


## Experimental

### 

#### Crystal data

C_40_H_36_N_8_O_2_
*M* *_r_* = 660.77
Monoclinic, 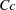

*a* = 13.148 (5) Å
*b* = 28.640 (7) Å
*c* = 9.757 (4) Å
β = 100.19 (2)°
*V* = 3616 (2) Å^3^
*Z* = 4
Mo *K*α radiation
μ = 0.08 mm^−1^
*T* = 293 K
0.6 × 0.5 × 0.4 mm

#### Data collection

Enraf–Nonius TurboCAD-4 diffractometer
3952 measured reflections
3787 independent reflections
2014 reflections with *I* > 2σ(*I*)
*R*_int_ = 0.039
3 standard reflections every 120 min intensity decay: −3%

#### Refinement

*R*[*F* ^2^ > 2σ(*F* ^2^)] = 0.038
*wR*(*F* ^2^) = 0.097
*S* = 0.91
3787 reflections
453 parameters
2 restraints
H-atom parameters constrained
Δρ_max_ = 0.15 e Å^−3^
Δρ_min_ = −0.15 e Å^−3^
Absolute structure: Flack (1983 ▶)
Absolute structure parameter: 0.00 (2)

### 

Data collection: *CAD-4 EXPRESS* (Enraf–Nonius, 1994 ▶); cell refinement: *CAD-4 EXPRESS*; data reduction: *XCAD4* (Harms & Wocadlo, 1996 ▶); program(s) used to solve structure: *SIR92* (Altomare *et al.*, 1994 ▶); program(s) used to refine structure: *SHELXL97* (Sheldrick, 2008 ▶); molecular graphics: *ORTEP-3 for Windows* (Farrugia, 2012 ▶); software used to prepare material for publication: *WinGX* publication routines (Farrugia, 2012 ▶).

## Supplementary Material

Crystal structure: contains datablock(s) I, global. DOI: 10.1107/S1600536813025671/ld2108sup1.cif

Structure factors: contains datablock(s) I. DOI: 10.1107/S1600536813025671/ld2108Isup2.hkl

Supplementary material file. DOI: 10.1107/S1600536813025671/ld2108Isup3.cml

Additional supplementary materials:  crystallographic information; 3D view; checkCIF report

## Figures and Tables

**Table 1:** Hydrogen-bond geometry (Å, °)

*D*—H⋯*A*	*D*—H	H⋯*A*	*D*⋯*A*	*D*—H⋯*A*
C15—H15⋯O2^i^	0.93	2.57	3.173 (6)	124 (3)
C36—H36⋯O1^ii^	0.93	2.55	3.409 (6)	153 (3)

Symmetry codes: (i) 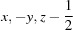; (ii) 
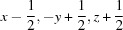
.

**Fig. 1.**
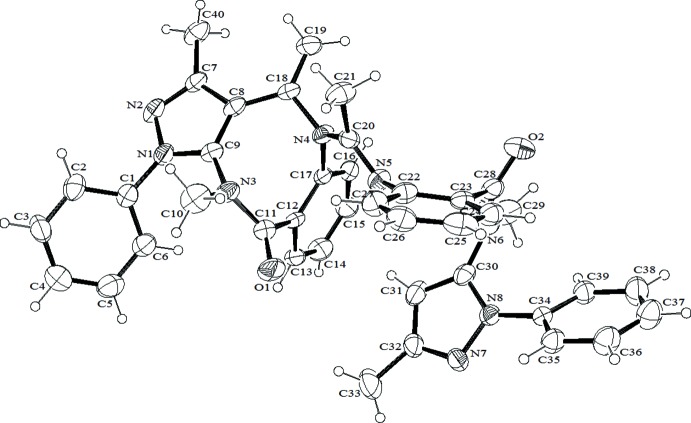
: The molecular structure of the title compound, showing atom-labeling scheme. Displacement ellipsoids for non-H atoms are at the 50% probability level.

**Fig. 2.**
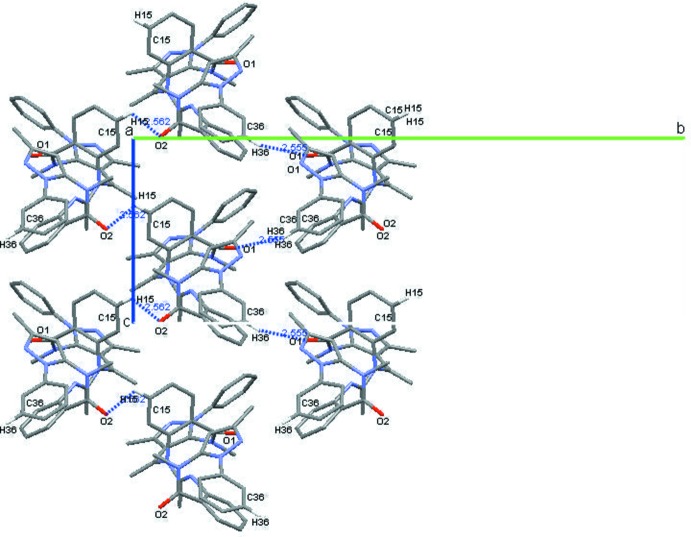
: Intermolecular interactions of the title compound, viewed along the a axis. Hydrogen bonds are shown as dashed lines. H atoms not involved in hydrogen bonding have been omitted.

## supplementary crystallographic information

### 1. Comment

We performed the Bischler-Napierlaski reaction (Plescia *et al.*, 1979) on 2-acetamido-*N*-methyl-*N*-(3-methyl-1-phenyl-1*H*-pyrazol-5-yl)benzamide by phosphorus oxychloride under reflux, obtaining an unexpected product related to a benzodiazocine system, whose corrected structure is now reported (Fig. 1). Single-crystal X-ray analysis on the reaction product allows to assign the formation of the title compound. The molecule crystallizes in a non-centrosymmetric space group: the possibility of the centrosymmetric space group *C*2/*c* was discounted by lack of a suitable solution and by the wholly satisfactory refinement in the space group *Cc*. The overall conformation of the molecular structure is determined by the central macrocycle formed by three fused cycles: a pyrazole, an eight-membered ring in the middle and a benzene. The central ring deviates from the ideal boat conformation because the bond between the *exo*-ethylene group and the adjacent nitrogen atom is twisted by 60 (1)° due to steric hindrances. The C12—C17 benzene and the pyrazole belonging to the tricyclic moiety are rather perpendicularly oriented (dihedral angle of 86 (1)°). The chain linked to N4 is characterized by a curled conformation, as defined by the torsion angles C20—N5—C22—C23 of -125 (1)° C22—C23—C28—N6 of -75 (1)° and C23—C28—N6—C30 of -10 (1)°. The benzene C22—C27 forms with the phenyl of the macrocycle a dihedral angle of 75 (1)°, while it makes a dihedral angle of 59 (1)° with the distal pyrazole unit. The latter moiety bears the C34—C39 ring quite rotated, as shown by the torsion angle N7—N8—C34—C35 of -41 (1)°. The phenyl C1—C6 linked to the fused pyrazole is rotated by 136 (1)°, as indicated by the torsion angle N2—N1—C1—C6. The molecular packing is stabilized by rather weak intermolecular Cπ—H···O type hydrogen bonds between C36—H36···O1i of 2.55 (1) Å and 153 (3)° [symmetry code: (i) *x* + 1/2, 3/2 - *y*, *z* - 1/2], and C15—H15ii···O2 at distance of 2.57 (1) Å, angle 124 (1)° [symmetry code: (ii) *x*, 2 - *y*, *z* + 1/2] (Fig. 2).

### 2. Experimental

A mixture of 2-acetamido-*N*-methyl-N-(3-methyl-1-phenyl-1*H*-pyrazol-5-yl)benzamide (14.5 mmoles) and phosphorus oxychloride (50 ml) was refluxed for 1 h. Excess phosphorus oxychloride was evaporated under reduced pressure and the reaction mixture was poured into crushed ice mixed with solid sodium bicarbonate and extracted with chloroform (3x150 ml): the organic layers were washed with water, dried (sodium sulfate) and concentrated under reduced pressure to dryness to give a residue, which was crystallized from ethanol affording the title compound (yield 1.75 g).

### 3. Refinement

All non-H-atoms were refined anisotropically. Hydrogen atoms were located by difference Fourier synthesis, except methyl and phenyl hydrogen atoms, that were introduced at calculated positions, in their described geometries and allowed to ride on the attached carbon atom with fixed isotropic thermal parameters 1.2Ueq and 1.5Ueq of the parent carbon atom for aromatic H-atoms and methyls H-atoms, respectively. For the methyls bound to the sp^2^-carbons, a rotating-group model was used. The crystal contains solvent accessible voids, however, no electron density peaks were found in chemically sensible positions for solvent molecules.

### Crystal data

**Table:**

C_40_H_36_N_8_O_2_	*F*(000) = 1392
*M**_r_* = 660.77	*D*_x_ = 1.214 Mg m^−^^3^
Monoclinic, *C**c*	Mo *K*α radiation, λ = 0.71073 Å
Hall symbol: C -2yc	Cell parameters from 25 reflections
*a* = 13.148 (5) Å	θ = 9–10°
*b* = 28.640 (7) Å	µ = 0.08 mm^−^^1^
*c* = 9.757 (4) Å	*T* = 293 K
β = 100.19 (2)°	Prism, colorless
*V* = 3616 (2) Å^3^	0.6 × 0.5 × 0.4 mm
*Z* = 4	

### Data collection

**Table:**

Enraf–Nonius TurboCAD-4 diffractometer	*R*_int_ = 0.039
Radiation source: fine-focus sealed tube	θ_max_ = 25.0°, θ_min_ = 2.5°
Graphite monochromator	*h* = −15→15
non–profiled ω/2θ scans	*k* = −1→34
3952 measured reflections	*l* = −1→11
3787 independent reflections	3 standard reflections every 120 min
2014 reflections with *I* > 2σ(*I*)	intensity decay: −3%

### Refinement

**Table:**

Refinement on *F*^2^	Secondary atom site location: difference Fourier map
Least-squares matrix: full	Hydrogen site location: inferred from neighbouring sites
*R*[*F*^2^ > 2σ(*F*^2^)] = 0.038	H-atom parameters constrained
*wR*(*F*^2^) = 0.097	*w* = 1/[σ^2^(*F*_o_^2^) + (0.0508*P*)^2^] where *P* = (*F*_o_^2^ + 2*F*_c_^2^)/3
*S* = 0.91	(Δ/σ)_max_ = 0.001
3787 reflections	Δρ_max_ = 0.15 e Å^−^^3^
453 parameters	Δρ_min_ = −0.15 e Å^−^^3^
2 restraints	Absolute structure: Flack, 1983
Primary atom site location: structure-invariant direct methods	Absolute structure parameter: 0.00 (2)

### Special details

**Table:**

Geometry. All e.s.d.'s (except the e.s.d. in the dihedral angle between two l.s. planes) are estimated using the full covariance matrix. The cell e.s.d.'s are taken into account individually in the estimation of e.s.d.'s in distances, angles and torsion angles; correlations between e.s.d.'s in cell parameters are only used when they are defined by crystal symmetry. An approximate (isotropic) treatment of cell e.s.d.'s is used for estimating e.s.d.'s involving l.s. planes.
Refinement. Refinement of *F*^2^ against ALL reflections. The weighted *R*-factor *wR* and goodness of fit *S* are based on *F*^2^, conventional *R*-factors *R* are based on *F*, with *F* set to zero for negative *F*^2^. The threshold expression of *F*^2^ > σ(*F*^2^) is used only for calculating *R*-factors(gt) *etc*. and is not relevant to the choice of reflections for refinement. *R*-factors based on *F*^2^ are statistically about twice as large as those based on *F*, and *R*- factors based on ALL data will be even larger.

### Fractional atomic coordinates and isotropic or equivalent isotropic displacement parameters (Å^2^)

**Table:**

	*x*	*y*	*z*	*U*_iso_*/*U*_eq_	
N4	0.6740 (2)	0.06127 (12)	0.7409 (4)	0.0483 (9)	
N3	0.7784 (3)	0.14236 (11)	0.6411 (4)	0.0540 (9)	
N2	0.9672 (2)	0.06694 (12)	0.5396 (4)	0.0574 (10)	
N5	0.5552 (3)	0.10524 (12)	0.8245 (4)	0.0520 (9)	
N8	0.2402 (2)	0.15797 (12)	0.7212 (4)	0.0476 (9)	
N7	0.2470 (3)	0.19540 (13)	0.6349 (4)	0.0582 (10)	
N6	0.3168 (2)	0.08240 (12)	0.7802 (4)	0.0523 (9)	
O1	0.6326 (2)	0.18574 (10)	0.5928 (4)	0.0804 (11)	
O2	0.3652 (3)	0.04921 (11)	0.9910 (4)	0.0782 (10)	
C30	0.3068 (3)	0.12341 (17)	0.6990 (5)	0.0527 (12)	
C22	0.5223 (3)	0.13466 (15)	0.9230 (5)	0.0543 (12)	
C11	0.6748 (3)	0.14808 (16)	0.5884 (5)	0.0568 (12)	
C8	0.8432 (3)	0.05827 (13)	0.6716 (5)	0.0481 (11)	
N1	0.9086 (2)	0.10631 (12)	0.5333 (4)	0.0536 (10)	
C34	0.1721 (3)	0.16005 (14)	0.8186 (4)	0.0445 (10)	
C23	0.4271 (3)	0.12559 (14)	0.9653 (4)	0.0479 (11)	
C12	0.6189 (3)	0.10572 (14)	0.5259 (5)	0.0437 (11)	
C17	0.6166 (3)	0.06490 (14)	0.6009 (4)	0.0412 (10)	
C20	0.6436 (3)	0.08519 (14)	0.8495 (5)	0.0474 (10)	
C1	0.9254 (3)	0.14421 (16)	0.4453 (5)	0.0555 (12)	
C18	0.7718 (3)	0.03634 (15)	0.7516 (5)	0.0486 (11)	
C39	0.1149 (3)	0.12067 (15)	0.8392 (5)	0.0580 (12)	
H39	0.1188	0.0938	0.7869	0.070*	
C38	0.0518 (4)	0.12229 (19)	0.9395 (6)	0.0759 (15)	
H38	0.0143	0.0959	0.9553	0.091*	
C13	0.5644 (3)	0.10817 (16)	0.3905 (5)	0.0550 (12)	
H13	0.5633	0.1360	0.3413	0.066*	
C28	0.3675 (3)	0.08260 (18)	0.9138 (5)	0.0560 (12)	
C24	0.3933 (3)	0.15488 (17)	1.0625 (5)	0.0621 (13)	
H24	0.3320	0.1483	1.0937	0.074*	
C27	0.5759 (4)	0.17442 (15)	0.9739 (5)	0.0620 (12)	
H27	0.6373	0.1816	0.9438	0.074*	
C35	0.1616 (3)	0.20001 (15)	0.8923 (5)	0.0608 (13)	
H35	0.1982	0.2267	0.8764	0.073*	
C7	0.9281 (3)	0.03763 (15)	0.6232 (5)	0.0526 (12)	
C9	0.8362 (3)	0.10225 (14)	0.6158 (5)	0.0470 (11)	
C6	0.8424 (4)	0.16392 (18)	0.3587 (5)	0.0696 (14)	
H6	0.7769	0.1509	0.3529	0.084*	
C4	0.9533 (4)	0.22134 (17)	0.2877 (6)	0.0733 (15)	
H4	0.9623	0.2483	0.2383	0.088*	
C15	0.5107 (3)	0.03006 (17)	0.4043 (5)	0.0605 (13)	
H15	0.4744	0.0043	0.3635	0.073*	
C40	0.9752 (3)	−0.00972 (15)	0.6485 (6)	0.0724 (15)	
H40A	1.0045	−0.0130	0.7454	0.109*	
H40B	1.0284	−0.0136	0.5936	0.109*	
H40C	0.9229	−0.0331	0.6229	0.109*	
C16	0.5615 (3)	0.02723 (16)	0.5397 (5)	0.0526 (12)	
H16	0.5587	−0.0002	0.5901	0.063*	
C10	0.8353 (4)	0.18250 (14)	0.7085 (6)	0.0808 (16)	
H10A	0.9064	0.1740	0.7392	0.121*	
H10B	0.8056	0.1920	0.7871	0.121*	
H10C	0.8315	0.2078	0.6433	0.121*	
C32	0.3164 (3)	0.18253 (19)	0.5574 (5)	0.0626 (13)	
C25	0.4510 (5)	0.19359 (16)	1.1125 (5)	0.0699 (14)	
H25	0.4283	0.2130	1.1773	0.084*	
C14	0.5123 (3)	0.07009 (18)	0.3284 (5)	0.0672 (13)	
H14	0.4787	0.0715	0.2363	0.081*	
C31	0.3563 (3)	0.13828 (19)	0.5951 (5)	0.0660 (13)	
H31	0.4063	0.1222	0.5573	0.079*	
C26	0.5413 (4)	0.20348 (17)	1.0672 (5)	0.0677 (14)	
H26	0.5791	0.2299	1.0997	0.081*	
C36	0.0979 (4)	0.20095 (17)	0.9889 (6)	0.0727 (15)	
H36	0.0913	0.2285	1.0372	0.087*	
C21	0.7165 (3)	0.08442 (19)	0.9866 (5)	0.0706 (14)	
H21A	0.7777	0.0674	0.9768	0.106*	
H21B	0.6836	0.0696	1.0553	0.106*	
H21C	0.7348	0.1158	1.0152	0.106*	
C3	1.0363 (4)	0.20026 (18)	0.3667 (6)	0.0766 (15)	
H3	1.1022	0.2120	0.3670	0.092*	
C37	0.0437 (4)	0.16234 (19)	1.0162 (6)	0.0788 (15)	
H37	0.0024	0.1630	1.0844	0.095*	
C19	0.7846 (4)	−0.00331 (16)	0.8214 (5)	0.0726 (14)	
H19A	0.7315	−0.0150	0.8630	0.087*	
H19B	0.8466	−0.0195	0.8290	0.087*	
C29	0.2745 (4)	0.03905 (16)	0.7128 (6)	0.0841 (17)	
H29A	0.2576	0.0180	0.7819	0.126*	
H29B	0.3250	0.0248	0.6660	0.126*	
H29C	0.2134	0.0460	0.6463	0.126*	
C2	1.0238 (3)	0.16203 (17)	0.4457 (6)	0.0699 (14)	
H2	1.0810	0.1479	0.4996	0.084*	
C33	0.3431 (4)	0.2152 (2)	0.4512 (6)	0.0978 (19)	
H33A	0.3016	0.2429	0.4488	0.147*	
H33B	0.3303	0.2004	0.3615	0.147*	
H33C	0.4149	0.2235	0.4748	0.147*	
C5	0.8557 (4)	0.20265 (19)	0.2807 (6)	0.0775 (15)	
H5	0.7994	0.2162	0.2236	0.093*	

### Atomic displacement parameters (Å^2^)

**Table:**

	*U*^11^	*U*^22^	*U*^33^	*U*^12^	*U*^13^	*U*^23^
N4	0.0416 (18)	0.061 (2)	0.040 (2)	0.0110 (16)	0.0016 (18)	0.0012 (19)
N3	0.048 (2)	0.039 (2)	0.072 (3)	0.0068 (16)	0.003 (2)	−0.010 (2)
N2	0.0343 (17)	0.053 (2)	0.082 (3)	0.0014 (18)	0.004 (2)	−0.020 (2)
N5	0.048 (2)	0.057 (2)	0.051 (2)	0.0060 (19)	0.0073 (19)	−0.006 (2)
N8	0.0431 (19)	0.053 (2)	0.046 (2)	0.0031 (18)	0.0067 (18)	0.0017 (19)
N7	0.054 (2)	0.064 (2)	0.057 (2)	−0.0069 (19)	0.010 (2)	0.016 (2)
N6	0.0445 (19)	0.049 (2)	0.061 (3)	0.0041 (17)	0.003 (2)	0.001 (2)
O1	0.069 (2)	0.0391 (17)	0.134 (3)	0.0175 (16)	0.019 (2)	0.004 (2)
O2	0.101 (3)	0.054 (2)	0.075 (2)	0.0096 (18)	0.004 (2)	0.014 (2)
C30	0.039 (2)	0.069 (3)	0.047 (3)	0.002 (2)	−0.003 (2)	−0.003 (3)
C22	0.062 (3)	0.049 (3)	0.051 (3)	0.012 (2)	0.005 (3)	−0.006 (2)
C11	0.051 (3)	0.054 (3)	0.065 (3)	0.006 (2)	0.009 (3)	0.010 (3)
C8	0.036 (2)	0.047 (3)	0.056 (3)	0.004 (2)	−0.007 (2)	−0.013 (2)
N1	0.039 (2)	0.049 (2)	0.072 (3)	−0.0002 (18)	0.006 (2)	−0.003 (2)
C34	0.040 (2)	0.044 (3)	0.051 (3)	0.005 (2)	0.010 (2)	0.006 (2)
C23	0.055 (3)	0.040 (2)	0.048 (3)	0.014 (2)	0.006 (2)	0.002 (2)
C12	0.034 (2)	0.045 (3)	0.050 (3)	0.0159 (19)	0.003 (2)	0.002 (2)
C17	0.032 (2)	0.049 (3)	0.042 (3)	0.0073 (19)	0.004 (2)	0.005 (2)
C20	0.050 (3)	0.044 (2)	0.047 (3)	0.000 (2)	0.004 (2)	0.008 (2)
C1	0.050 (3)	0.055 (3)	0.061 (3)	−0.001 (2)	0.010 (3)	−0.008 (3)
C18	0.041 (2)	0.047 (2)	0.053 (3)	0.011 (2)	−0.003 (2)	0.002 (2)
C39	0.058 (3)	0.048 (3)	0.071 (3)	0.004 (2)	0.021 (3)	−0.002 (3)
C38	0.067 (3)	0.080 (4)	0.085 (4)	−0.008 (3)	0.027 (3)	0.002 (3)
C13	0.051 (3)	0.060 (3)	0.055 (3)	0.019 (2)	0.014 (3)	0.017 (3)
C28	0.051 (3)	0.058 (3)	0.060 (3)	0.017 (2)	0.015 (3)	0.004 (3)
C24	0.065 (3)	0.070 (3)	0.053 (3)	0.020 (3)	0.014 (3)	0.004 (3)
C27	0.067 (3)	0.056 (3)	0.064 (3)	0.005 (3)	0.015 (3)	−0.009 (3)
C35	0.058 (3)	0.048 (3)	0.079 (4)	0.004 (2)	0.020 (3)	−0.004 (3)
C7	0.035 (2)	0.044 (2)	0.073 (3)	0.003 (2)	−0.005 (2)	−0.009 (3)
C9	0.045 (2)	0.040 (3)	0.052 (3)	0.003 (2)	−0.003 (2)	−0.008 (2)
C6	0.055 (3)	0.080 (4)	0.072 (4)	0.006 (3)	0.007 (3)	0.006 (3)
C4	0.094 (4)	0.062 (3)	0.065 (4)	−0.004 (3)	0.017 (3)	−0.005 (3)
C15	0.054 (3)	0.067 (3)	0.054 (3)	−0.009 (2)	−0.009 (3)	0.002 (3)
C40	0.053 (3)	0.061 (3)	0.101 (4)	0.016 (2)	0.007 (3)	−0.017 (3)
C16	0.042 (2)	0.061 (3)	0.055 (3)	−0.001 (2)	0.007 (2)	0.006 (3)
C10	0.083 (3)	0.052 (3)	0.102 (4)	−0.006 (3)	0.001 (3)	−0.030 (3)
C32	0.046 (3)	0.088 (4)	0.052 (3)	−0.007 (3)	0.005 (3)	0.017 (3)
C25	0.106 (4)	0.049 (3)	0.049 (3)	0.020 (3)	0.000 (3)	−0.008 (3)
C14	0.062 (3)	0.081 (4)	0.052 (3)	0.004 (3)	−0.008 (3)	−0.008 (3)
C31	0.044 (2)	0.105 (4)	0.050 (3)	0.012 (3)	0.010 (2)	0.002 (3)
C26	0.087 (4)	0.052 (3)	0.063 (3)	0.003 (3)	0.010 (3)	−0.001 (3)
C36	0.072 (3)	0.060 (3)	0.090 (4)	0.004 (3)	0.024 (3)	−0.016 (3)
C21	0.072 (3)	0.089 (3)	0.046 (3)	0.025 (3)	−0.001 (3)	−0.006 (3)
C3	0.074 (3)	0.085 (4)	0.070 (4)	−0.028 (3)	0.011 (3)	0.015 (3)
C37	0.074 (3)	0.081 (4)	0.087 (4)	0.008 (3)	0.029 (3)	−0.012 (4)
C19	0.068 (3)	0.072 (3)	0.077 (4)	0.027 (3)	0.013 (3)	0.021 (3)
C29	0.080 (3)	0.066 (3)	0.097 (4)	0.006 (3)	−0.009 (3)	−0.016 (3)
C2	0.049 (3)	0.081 (3)	0.075 (4)	−0.015 (3)	−0.001 (3)	−0.008 (3)
C33	0.083 (4)	0.140 (5)	0.073 (4)	−0.014 (4)	0.022 (3)	0.039 (4)
C5	0.073 (4)	0.089 (4)	0.071 (4)	0.022 (3)	0.014 (3)	0.005 (3)

### Geometric parameters (Å, º)

**Table:**

N4—C20	1.380 (5)	C24—H24	0.9300
N4—C17	1.443 (5)	C27—C26	1.370 (6)
N4—C18	1.458 (5)	C27—H27	0.9300
N3—C11	1.378 (5)	C35—C36	1.368 (7)
N3—C9	1.422 (5)	C35—H35	0.9300
N3—C10	1.463 (5)	C7—C40	1.493 (6)
N2—C7	1.335 (6)	C6—C5	1.374 (7)
N2—N1	1.361 (4)	C6—H6	0.9300
N5—C20	1.281 (5)	C4—C3	1.361 (7)
N5—C22	1.402 (5)	C4—C5	1.380 (7)
N8—C30	1.364 (5)	C4—H4	0.9300
N8—N7	1.375 (4)	C15—C14	1.367 (6)
N8—C34	1.418 (5)	C15—C16	1.374 (6)
N7—C32	1.336 (6)	C15—H15	0.9300
N6—C28	1.355 (6)	C40—H40A	0.9600
N6—C30	1.410 (6)	C40—H40B	0.9600
N6—C29	1.467 (6)	C40—H40C	0.9600
O1—C11	1.217 (5)	C16—H16	0.9300
O2—C28	1.221 (5)	C10—H10A	0.9600
C30—C31	1.366 (6)	C10—H10B	0.9600
C22—C27	1.384 (6)	C10—H10C	0.9600
C22—C23	1.410 (6)	C32—C31	1.396 (6)
C11—C12	1.492 (6)	C32—C33	1.484 (7)
C8—C9	1.369 (5)	C25—C26	1.368 (7)
C8—C7	1.416 (5)	C25—H25	0.9300
C8—C18	1.464 (6)	C14—H14	0.9300
N1—C9	1.356 (5)	C31—H31	0.9300
N1—C1	1.426 (6)	C26—H26	0.9300
C34—C35	1.371 (6)	C36—C37	1.367 (6)
C34—C39	1.389 (6)	C36—H36	0.9300
C23—C24	1.397 (6)	C21—H21A	0.9600
C23—C28	1.498 (6)	C21—H21B	0.9600
C12—C17	1.382 (5)	C21—H21C	0.9600
C12—C13	1.389 (6)	C3—C2	1.365 (6)
C17—C16	1.375 (5)	C3—H3	0.9300
C20—C21	1.501 (6)	C37—H37	0.9300
C1—C6	1.378 (6)	C19—H19A	0.9300
C1—C2	1.389 (6)	C19—H19B	0.9300
C18—C19	1.320 (5)	C29—H29A	0.9600
C39—C38	1.391 (7)	C29—H29B	0.9600
C39—H39	0.9300	C29—H29C	0.9600
C38—C37	1.384 (7)	C2—H2	0.9300
C38—H38	0.9300	C33—H33A	0.9600
C13—C14	1.371 (6)	C33—H33B	0.9600
C13—H13	0.9300	C33—H33C	0.9600
C24—C25	1.383 (6)	C5—H5	0.9300
			
C20—N4—C17	121.2 (3)	C8—C9—N3	132.4 (4)
C20—N4—C18	124.1 (3)	C5—C6—C1	120.3 (5)
C17—N4—C18	114.2 (3)	C5—C6—H6	119.8
C11—N3—C9	123.7 (4)	C1—C6—H6	119.8
C11—N3—C10	117.9 (3)	C3—C4—C5	120.1 (5)
C9—N3—C10	117.8 (4)	C3—C4—H4	120.0
C7—N2—N1	105.8 (3)	C5—C4—H4	120.0
C20—N5—C22	121.2 (4)	C14—C15—C16	121.2 (4)
C30—N8—N7	110.8 (4)	C14—C15—H15	119.4
C30—N8—C34	129.5 (4)	C16—C15—H15	119.4
N7—N8—C34	119.6 (3)	C7—C40—H40A	109.5
C32—N7—N8	104.8 (3)	C7—C40—H40B	109.5
C28—N6—C30	121.2 (4)	H40A—C40—H40B	109.5
C28—N6—C29	121.2 (4)	C7—C40—H40C	109.5
C30—N6—C29	117.6 (4)	H40A—C40—H40C	109.5
N8—C30—C31	107.2 (4)	H40B—C40—H40C	109.5
N8—C30—N6	120.9 (4)	C15—C16—C17	120.0 (4)
C31—C30—N6	131.9 (4)	C15—C16—H16	120.0
C27—C22—N5	122.8 (4)	C17—C16—H16	120.0
C27—C22—C23	117.9 (4)	N3—C10—H10A	109.5
N5—C22—C23	119.1 (4)	N3—C10—H10B	109.5
O1—C11—N3	121.3 (4)	H10A—C10—H10B	109.5
O1—C11—C12	122.7 (4)	N3—C10—H10C	109.5
N3—C11—C12	116.0 (4)	H10A—C10—H10C	109.5
C9—C8—C7	104.7 (4)	H10B—C10—H10C	109.5
C9—C8—C18	127.0 (4)	N7—C32—C31	111.3 (4)
C7—C8—C18	128.0 (4)	N7—C32—C33	119.4 (5)
C9—N1—N2	110.7 (4)	C31—C32—C33	129.2 (5)
C9—N1—C1	128.5 (4)	C26—C25—C24	120.4 (5)
N2—N1—C1	120.7 (4)	C26—C25—H25	119.8
C35—C34—C39	119.7 (4)	C24—C25—H25	119.8
C35—C34—N8	121.4 (4)	C15—C14—C13	118.9 (5)
C39—C34—N8	119.0 (4)	C15—C14—H14	120.6
C24—C23—C22	119.6 (4)	C13—C14—H14	120.6
C24—C23—C28	120.7 (4)	C30—C31—C32	105.8 (4)
C22—C23—C28	119.5 (4)	C30—C31—H31	127.1
C17—C12—C13	119.4 (4)	C32—C31—H31	127.1
C17—C12—C11	121.8 (4)	C25—C26—C27	119.9 (5)
C13—C12—C11	118.8 (4)	C25—C26—H26	120.1
C16—C17—C12	119.6 (4)	C27—C26—H26	120.1
C16—C17—N4	120.1 (4)	C37—C36—C35	121.5 (5)
C12—C17—N4	120.3 (4)	C37—C36—H36	119.3
N5—C20—N4	116.9 (4)	C35—C36—H36	119.3
N5—C20—C21	126.3 (4)	C20—C21—H21A	109.5
N4—C20—C21	116.8 (4)	C20—C21—H21B	109.5
C6—C1—C2	119.2 (5)	H21A—C21—H21B	109.5
C6—C1—N1	119.4 (4)	C20—C21—H21C	109.5
C2—C1—N1	121.4 (4)	H21A—C21—H21C	109.5
C19—C18—N4	119.2 (4)	H21B—C21—H21C	109.5
C19—C18—C8	127.6 (4)	C4—C3—C2	120.7 (5)
N4—C18—C8	113.1 (3)	C4—C3—H3	119.6
C34—C39—C38	118.7 (5)	C2—C3—H3	119.6
C34—C39—H39	120.7	C36—C37—C38	118.2 (5)
C38—C39—H39	120.7	C36—C37—H37	120.9
C37—C38—C39	121.4 (5)	C38—C37—H37	120.9
C37—C38—H38	119.3	C18—C19—H19A	120.0
C39—C38—H38	119.3	C18—C19—H19B	120.0
C14—C13—C12	120.9 (4)	H19A—C19—H19B	120.0
C14—C13—H13	119.6	N6—C29—H29A	109.5
C12—C13—H13	119.6	N6—C29—H29B	109.5
O2—C28—N6	121.9 (5)	H29A—C29—H29B	109.5
O2—C28—C23	120.4 (5)	N6—C29—H29C	109.5
N6—C28—C23	117.8 (4)	H29A—C29—H29C	109.5
C25—C24—C23	120.0 (5)	H29B—C29—H29C	109.5
C25—C24—H24	120.0	C3—C2—C1	119.9 (5)
C23—C24—H24	120.0	C3—C2—H2	120.1
C26—C27—C22	122.1 (5)	C1—C2—H2	120.1
C26—C27—H27	119.0	C32—C33—H33A	109.5
C22—C27—H27	119.0	C32—C33—H33B	109.5
C36—C35—C34	120.5 (4)	H33A—C33—H33B	109.5
C36—C35—H35	119.7	C32—C33—H33C	109.5
C34—C35—H35	119.7	H33A—C33—H33C	109.5
N2—C7—C8	110.6 (4)	H33B—C33—H33C	109.5
N2—C7—C40	118.6 (4)	C6—C5—C4	119.7 (5)
C8—C7—C40	130.7 (4)	C6—C5—H5	120.2
N1—C9—C8	108.0 (3)	C4—C5—H5	120.2
N1—C9—N3	119.1 (4)		
			
C30—N8—N7—C32	1.8 (4)	C17—C12—C13—C14	−2.9 (6)
C34—N8—N7—C32	−180.0 (4)	C11—C12—C13—C14	179.7 (4)
N7—N8—C30—C31	−1.1 (5)	C30—N6—C28—O2	170.1 (4)
C34—N8—C30—C31	−179.1 (4)	C29—N6—C28—O2	−12.9 (6)
N7—N8—C30—N6	177.1 (3)	C30—N6—C28—C23	−9.6 (5)
C34—N8—C30—N6	−1.0 (6)	C29—N6—C28—C23	167.4 (4)
C28—N6—C30—N8	−74.1 (5)	C24—C23—C28—O2	−70.1 (5)
C29—N6—C30—N8	108.7 (5)	C22—C23—C28—O2	105.2 (5)
C28—N6—C30—C31	103.5 (6)	C24—C23—C28—N6	109.7 (5)
C29—N6—C30—C31	−73.6 (6)	C22—C23—C28—N6	−75.0 (5)
C20—N5—C22—C27	59.3 (6)	C22—C23—C24—C25	2.6 (6)
C20—N5—C22—C23	−125.3 (4)	C28—C23—C24—C25	177.9 (4)
C9—N3—C11—O1	−167.6 (5)	N5—C22—C27—C26	178.2 (4)
C10—N3—C11—O1	2.7 (7)	C23—C22—C27—C26	2.8 (6)
C9—N3—C11—C12	12.1 (6)	C39—C34—C35—C36	2.1 (7)
C10—N3—C11—C12	−177.5 (4)	N8—C34—C35—C36	−178.0 (4)
C7—N2—N1—C9	2.1 (4)	N1—N2—C7—C8	0.1 (4)
C7—N2—N1—C1	−176.2 (4)	N1—N2—C7—C40	178.7 (4)
C30—N8—C34—C35	137.3 (5)	C9—C8—C7—N2	−2.1 (5)
N7—N8—C34—C35	−40.6 (5)	C18—C8—C7—N2	172.4 (4)
C30—N8—C34—C39	−42.8 (6)	C9—C8—C7—C40	179.5 (4)
N7—N8—C34—C39	139.3 (4)	C18—C8—C7—C40	−6.0 (7)
C27—C22—C23—C24	−3.9 (6)	N2—N1—C9—C8	−3.5 (4)
N5—C22—C23—C24	−179.5 (4)	C1—N1—C9—C8	174.6 (4)
C27—C22—C23—C28	−179.3 (4)	N2—N1—C9—N3	169.9 (3)
N5—C22—C23—C28	5.1 (6)	C1—N1—C9—N3	−12.0 (6)
O1—C11—C12—C17	−123.3 (5)	C7—C8—C9—N1	3.3 (4)
N3—C11—C12—C17	56.9 (5)	C18—C8—C9—N1	−171.3 (4)
O1—C11—C12—C13	54.1 (6)	C7—C8—C9—N3	−168.9 (4)
N3—C11—C12—C13	−125.7 (4)	C18—C8—C9—N3	16.6 (7)
C13—C12—C17—C16	0.8 (5)	C11—N3—C9—N1	110.1 (5)
C11—C12—C17—C16	178.2 (4)	C10—N3—C9—N1	−60.2 (5)
C13—C12—C17—N4	179.0 (3)	C11—N3—C9—C8	−78.4 (6)
C11—C12—C17—N4	−3.7 (5)	C10—N3—C9—C8	111.3 (5)
C20—N4—C17—C16	−110.6 (4)	C2—C1—C6—C5	−4.3 (7)
C18—N4—C17—C16	77.7 (4)	N1—C1—C6—C5	175.1 (4)
C20—N4—C17—C12	71.2 (5)	C14—C15—C16—C17	−0.9 (7)
C18—N4—C17—C12	−100.5 (4)	C12—C17—C16—C15	1.1 (6)
C22—N5—C20—N4	−172.8 (4)	N4—C17—C16—C15	−177.1 (4)
C22—N5—C20—C21	8.5 (6)	N8—N7—C32—C31	−1.8 (5)
C17—N4—C20—N5	9.5 (6)	N8—N7—C32—C33	−179.9 (4)
C18—N4—C20—N5	−179.7 (4)	C23—C24—C25—C26	0.1 (7)
C17—N4—C20—C21	−171.6 (4)	C16—C15—C14—C13	−1.2 (7)
C18—N4—C20—C21	−0.8 (6)	C12—C13—C14—C15	3.1 (6)
C9—N1—C1—C6	−46.4 (6)	N8—C30—C31—C32	−0.1 (5)
N2—N1—C1—C6	131.5 (4)	N6—C30—C31—C32	−177.9 (4)
C9—N1—C1—C2	133.1 (5)	N7—C32—C31—C30	1.2 (5)
N2—N1—C1—C2	−49.0 (6)	C33—C32—C31—C30	179.1 (5)
C20—N4—C18—C19	72.7 (5)	C24—C25—C26—C27	−1.3 (7)
C17—N4—C18—C19	−115.9 (5)	C22—C27—C26—C25	−0.2 (7)
C20—N4—C18—C8	−111.4 (4)	C34—C35—C36—C37	0.6 (7)
C17—N4—C18—C8	60.0 (4)	C5—C4—C3—C2	−3.5 (8)
C9—C8—C18—C19	−170.2 (5)	C35—C36—C37—C38	−2.3 (8)
C7—C8—C18—C19	16.6 (7)	C39—C38—C37—C36	1.3 (8)
C9—C8—C18—N4	14.3 (6)	C4—C3—C2—C1	0.2 (8)
C7—C8—C18—N4	−158.9 (4)	C6—C1—C2—C3	3.7 (7)
C35—C34—C39—C38	−3.0 (6)	N1—C1—C2—C3	−175.8 (4)
N8—C34—C39—C38	177.1 (4)	C1—C6—C5—C4	1.1 (7)
C34—C39—C38—C37	1.3 (7)	C3—C4—C5—C6	2.8 (8)

### Hydrogen-bond geometry (Å, º)

**Table:**

*D*—H···*A*	*D*—H	H···*A*	*D*···*A*	*D*—H···*A*
C15—H15···O2^i^	0.93	2.57	3.173 (6)	124 (3)
C36—H36···O1^ii^	0.93	2.55	3.409 (6)	153 (3)

Symmetry codes: (i) *x*, −*y*, *z*−1/2; (ii) *x*−1/2, −*y*+1/2, *z*+1/2.
